# Atractylenolide I Inhibits Nicotine-Induced Macrophage Pyroptosis and Alleviates Atherogenesis by Suppressing the TLR4/ROS/TXNIP/NLRP3 Pathway

**DOI:** 10.3390/metabo15050329

**Published:** 2025-05-15

**Authors:** Huan-Huan Li, Xian Liu, Yu-Ping Wang, Xi Xu, Lin Zhu, Wei Zhang, Kun Ren

**Affiliations:** 1 College of Traditional Chinese Medicine, Anhui University of Chinese Medicine, Hefei 230012, China; 2 College of Nursing, Anhui University of Chinese Medicine, Hefei 230012, China; salt0401@stu.ahtcm.edu.cn (X.L.); yupingwang@stu.ahtcm.edu.cn (Y.-P.W.);; 3 College of Pharmacy, Anhui University of Chinese Medicine, Hefei 230012, China; zhulin@ahtcm.edu.cn; 4 Anhui Province Key Laboratory of Bioactive Natural Products, Hefei 230012, China; 5 Laboratory of Geriatric Nursing and Health, Anhui University of Traditional Chinese Medicine, Hefei 230012, China

**Keywords:** Atractylenolide I, nicotine, macrophage, pyroptosis, atherosclerosis

## Abstract

**Background/Objectives:** Studies have shown that Atractylenolide I (AT-I) can exert anti-inflammatory and anti-oxidative effects, protecting against the development of various kinds of cardiovascular diseases. However, whether AT-I prevents nicotine-induced atherogenesis is unknown. This study was designed to explore the effects of AT-I on nicotine-induced macrophage pyroptosis and the progression of atherosclerosis. **Methods:** RT-qPCR and Western blot were used to detect the mRNA and protein levels of TXNIP and pyroptosis-related factors in THP-1-derived macrophages. ELISA was used to detect the secretion of pro-inflammatory cytokines. Hoechst/PI double-staining assay was used to assess plasma membrane integrity. The ROS assay kit, LDH release assay kit, and caspase-1 activity assay kit were used to detect ROS production, LDH release, and caspase-1 activity. Oil Red O, HE, and Masson staining were used to evaluate lipid accumulation, lesion size, and plaque stability in HFD-fed apoE^−/−^ mice. **Results:** AT-I treatment significantly decreased pyroptosis-related factors expression, disrupted plasma membrane integrity, and down-regulated pro-inflammatory cytokines secretion, thereby inhibiting nicotine-induced pyroptosis of THP-1-derived macrophages. In addition, AT-I decreased ROS production and the expression of TLR4 and TXNIP. Lentivirus overexpression of TLR4 or TXNIP, or pre-treatment with ROS agonist, mainly reversed the anti-pyroptotic effects of AT-I in nicotine-treated THP-1-derived macrophages. Additionally, administering AT-I to HFD-fed apoE^−/−^ mice markedly decreased nicotine-induced up-regulation of pyroptosis-related proteins in the aortas. Enzymatic methods and ELISA assay suggested that AT-I improved dyslipidemia and inflammation in vivo. Oil Red O, HE, and Masson staining showed that AT-I alleviated lipid accumulation, decreased plaque size, and increased plaque stability. **Conclusions:** Taken together, AT-I can be regarded as a potential phytomedicine that protects against macrophage pyroptosis and atherosclerosis triggered by nicotine.

## 1. Introduction

Atherosclerotic cardiovascular disease (ASCVD) is the foremost cause of death worldwide [1]. The main cause of ASCVD is atherosclerosis, which is marked by significant lipid accumulation and ongoing chronic inflammation in the blood vessel walls [2]. Studies have shown that antagonizing interleukin-1β (IL-1β) could reduce primary clinical endpoints and lower cardiovascular mortality in patients with ASCVD [3]. The deletion of IL-1β led to a 33% reduction in total atherosclerotic lesions in high-fat diet (HFD)-fed apolipoprotein E deficient (apoE^−^^/−^) mice, demonstrating the effectiveness of anti-inflammatory treatment in improving atherosclerosis [4]. The development of atherosclerosis is closely related to programmed cell death within plaques [5]. Pyroptosis, a form of programmed cell death (PCD), is driven by the activation of inflammatory caspases and the formation of membrane pores mediated by gasdermin (GSDM), inducing inflammation [6]. Macrophage pyroptosis is detrimental to the initiation and progression of atherosclerosis. Compared to healthy arteries, levels of pyroptosis-associated proteins are significantly up-regulated in mouse carotid artery plaques [7]. Fan et al. reported that GSDMD is markedly activated during atherosclerosis and is principally expressed in plaque macrophages. Knockout of GSDMD attenuates aortic pyroptosis and decreases atherosclerotic lesions in HFD-fed apoE^−/−^ mice [8]. In addition, many food extracts [9] and Chinese herbal medicines [10,11] are demonstrated to mitigate the development of atherosclerosis by suppressing macrophage pyroptosis. Thus, a thorough investigation into the regulatory mechanisms of macrophage pyroptosis can be beneficial in slowing down atherogenesis.

**Figure 1:**
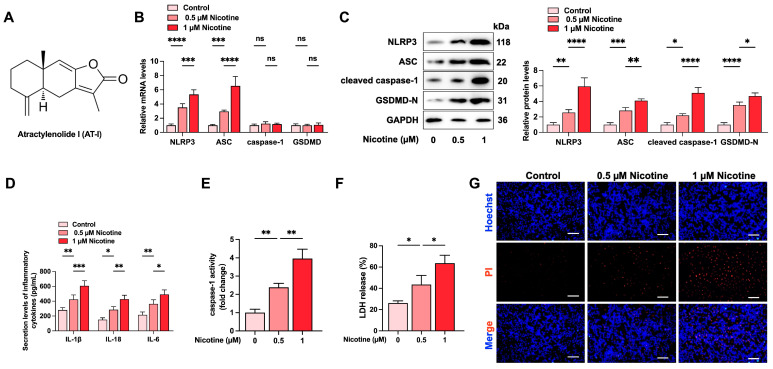
Nicotine induces pyroptosis of THP-1-derived macrophages. Cells were treated with 0 µM, 0.5 µM, and 1 µM nicotine for 24 h. (**A**) Chemical structure of AT-I. (**B**) Analysis of mRNA levels of pyroptosis-related factors using RT-qPCR. (**C**) Western blot analysis of the expression of pyroptosis-related proteins. (**D**) Evaluation of IL-1β, IL-6, and IL-18 secretion levels by ELISA; (**E**) Detection of caspase-1 activity. (**F**) Evaluation of LDH release. (**G**) Hoechst/PI staining. Scale bar = 20 μm. * *p* < 0.05, ** *p* < 0.01, *** *p* < 0.001, **** *p* < 0.0001, ns, not significant. *n* = 3.

Smoking is a significant cause of preventable death. It is estimated that smoking-associated diseases kill 7 million people annually worldwide, and this number is projected to reach 8 million [12,13]. Nicotine, the main ingredient of tobacco, can induce thrombosis, angiogenesis, abnormal vascular growth, dyslipidemia, and vascular inflammation, resulting in the progression of atherosclerosis [14]. Atractylenolide I (AT-I, Figure 1A) is a bioactive compound isolated from the Chinese herb *Atractylodes macrocephala* Koidz (known as Baizhu in China) [15,16]. AT-I was well-established as an anti-cancer agent. It can induce the apoptosis of many cancer cell types by increasing reactive oxygen species (ROS) generation and inhibiting the JAK2/STAT3 signaling pathway, including leukemia cells [17], melanoma cells [18], and colorectal cancer cells [19]. AT-I can also suppress the proliferation of cancer cells by suppressing the PI3K/AKT pathway [20]. In addition, AT-I has anti-oxidant and anti-coagulant properties, exerting protective effects on many kinds of CVDs, such as ischemia/reperfusion injury [21], abdominal aortic aneurysm formation [22], and cardiac hypertrophy [23]. Moreover, AT-I was found effective in the treatment of inflammatory illness. In the LPS-induced acute lung injury mouse model, administering AT-I significantly down-regulated the production of many inflammatory cytokines [24]. Treatment of rat vascular smooth muscle cells with AT-I increased heme oxygenase-1 (HO-1) expression and decreased the production of nitric oxide (NO), tumor necrosis factor-alpha (TNF-α), and interleukin-6 (IL-6) induced by ox-LDL [25]. Furthermore, AT-I has potent inhibitory effects on macrophage inflammation. AT-I can dose-dependently inhibit the production of TNF-α, NO, IL-6, and IL-1β in LPS-stimulated mouse peritoneal macrophages [26]. Treatment of mouse RAW 264.7 macrophages with AT-I inhibited LPS-induced M1 polarization by inhibiting the activation of the PARP1/NLRP3 signaling pathway, suppressing oxidative stress, and cardiomyocyte injury [27]. Likewise, AT-I can suppress LPS-induced TNF-α and NO production in mouse peritoneal macrophages [28]. However, whether AT-I inhibits nicotine-induced atherogenesis by suppressing macrophage pyroptosis remains unclear.

Mao et al. found that nicotine intake by HFD-apoE^−/−^ mice caused higher TXNIP expression in peripheral blood mononuclear cells (PBMCs) and elevated pro-inflammatory levels in the serum. Nicotine treatment promoted lipid phagocytosis and pyroptosis in bone marrow-derived macrophages (BMDMs) by activating the ROS/TXNIP/NLRP3 signaling [29]. Many bioactive herbal extracts provide protection via the ROS/TXNIP/NLRP3 signaling pathway. Piperlongumine can inhibit the proliferation, migration, invasion, and colony formation of squamous cells and alleviate the tumorigenesis of squamous cell carcinoma by inhibiting NF-E2-related factor 2 (NRF2) and promoting ROS/TXNIP/NLRP3-mediated pyroptosis [30]. Artemisinin can inhibit the activation of the ROS/TXNIP/NLRP3/caspase-1 pathway and suppress neuronal pyroptosis [31]. Decursinol angelate can ameliorate epithelial cell pyroptosis by inhibiting the ROS/TXNIP/NLRP3 pathway, mitigating inflammatory bowel disease [32]. It is still unknown, nevertheless, whether AT-I inhibits nicotine-induced macrophage pyroptosis by modulating this pathway.

Here, we found that AT-I significantly improved lipid profile and reduced inflammation, which contributed to the alleviation of atherosclerosis in nicotine-treated apoE^−/−^ mice. Importantly, AT-I decreases TLR4 levels and subsequent ROS/TXNIP/NLRP3 signaling pathway activation, thereby mitigating nicotine-induced pyroptosis of THP-1-derived macrophages. AT-I can be regarded as a potential phytomedicine for treating nicotine-mediated ASCVDs.

## 2. Materials and Methods

### 2.1. Cell Culture and Treatment

Human THP-1 monocytes were bought from Pricella (#CL-0233, Wuhan, China). They were maintained in RPMI 1640 medium supplemented with 10% FBS and 1% antibiotics under standard conditions (37 °C, 5% CO_2_). Cells were incubated with 160 nM PMA for differentiation into adherent macrophages for 48 h. Before experiments, cells were maintained without serum for 8 h for synchronization. To increase ROS production, cells were incubated with 10 µM DMNQ (ROS agonist, #HY-121026, MedChemExpress) for 6 h. For the overexpression of TLR4 and TXNIP, macrophages were transfected with lentiviral empty vector (LV-NC), a lentiviral vector expressing TLR4 (LV-TXNIP), or LV-TXNIP (Genechem, Shanghai) at 100 multiplicity of infection (MOI) using 8 µg/mL polybrene (#TR-1003, Sigma-Aldrich, St. Louis, MO, USA) for 72 h at 37 °C, followed by Western blot analysis.

### 2.2. RNA Isolation and RT-qPCR

After treating the macrophages as indicated, total intracellular RNA was extracted using the TRIzol reagent, and the purity was assessed with a Nanodrop 3000 spectrophotometer. Approximately 2 µg cDNA was synthesized using the TaqMan Reverse Transcription Kit (#N8080234, Invitrogen, Waltham, MA, USA). The PCR process was conducted on the ABI 7500 Real-Time PCR System using an UltraSYBR Mixture kit (#CW0957M, CWBIO). The PCR program comprised 30 s at 95 °C for pre-denaturation, 40 cycles of 10 s at 95 °C, and 30 s at 60 °C. All primers (listed in Supplementary Table S1) were designed and synthesized by Sangon Biotech. Each sample was assayed with at least three replicates, and the 2^−Δ∆Ct^ method was used to quantify gene expression, as previously described [33,34,35,36,37]. GAPDH was used as the endogenous control.

### 2.3. Western Blot Analysis

Total proteins were extracted using a mixture of RIPA buffer and PMSF (94:6). Protein solutions were obtained through centrifugation at 12,000 rpm for 10 min, and the concentrations were measured using a BCA assay kit (#PC0020, Solarbio, Beijing, China). The SDS-PAGE loading buffers (#CW0028S, CWBIO, Cambridge, MA, USA) were added to the protein solution at a ratio of 4:1, and the mixture was vortexed and boiled for 8 min. Protein lysates were separated using a 10% SDS-PAGE gel (approximately 20 µg proteins per lane) for 1.5 h at 110 V. Then, proteins were transferred to PVDF membranes (#IPVH00010, Millipore, Burlington, MA, USA) at a constant current of 260 mA for 2 h, followed by soaking with primary antibodies (1:1000) against TLR4 (#19811-1-AP, Proteintech, Wuhan, China), TXNIP (#18243-1-AP, Proteintech), NLRP3 (#27458-1-AP, Proteintech), cleaved-caspase-1 (Asp296) (#AF4005, Affinity Biosciences, Jiangsu, China), ASC (#10500-1-AP, Proteintech), GSDMD-N (#PU224937, ABmart, Shanhai, China), and GAPDH (#10494-1-AP, Proteintech) overnight at 4 °C. After rinses in TBS-T thrice, membranes were further incubated with a HRP-conjugated antibody (1:5000, Proteintech, #10500-1-AP) for 4 h at 4 °C. The ECL Substrate (Merck, #WBULP-10ML) was used to visualize protein bands, and the Image Pro Plus software was utilized to quantify protein expression [38].

### 2.4. ELISA Assay

THP-1-derived macrophages and HFD-fed apoE^−/−^ mice were treated accordingly, and culture media and plasma samples were collected from each group. Human IL-18 (#DL180), IL-1β (#DLB50), and IL-6 (#D6050B) and mouse TNF-α (#MTA00B), IL-1β (#MLB00C), and IL-6 (#M6000B) were detected strictly per the manual instructions of the respective ELISA kits (R&D Systems). Absorbance at 450 nm was monitored using a microplate reader.

### 2.5. Detection of Caspase-1 Activity

The caspase-1 activity in THP-1-derived macrophages was assessed using the caspase-1 activity assay kit (#C1101, Beyotime) [38]. Cells in 6-well plates were incubated with 200 µL lysis buffer for 30 min on ice. Cell debris was then collected, transferred into a 1.5 mL EP tube, and centrifuged. The supernatants were supplemented with 10 μL AcYVAD-pNA for 2 h at room temperature. Absorbance at 405 nm was monitored, and the standard pNA (yellow) curve was plotted. Caspase-1 activity was normalized to the control group.

### 2.6. LDH Release Assay

LDH release assay was conducted using an LDH cytotoxicity assay kit (Beyotime, #C0016), as described previously [39]. In brief, after various treatments, THP-1-derived macrophages were planted in 96-well plates with 100 µL RPMI complete medium per well. Each well was supplemented with 10 µL LDH release solution and maintained for 12 h at 37 °C. An aliquot of 50 μL medium or cell lysates was mixed with 60 µL LDH working solution for 30 min at 4 °C. Absorbance at 490 nm was recorded. The percentage of LDH secretion was evaluated as follows: [the absorbance of the supernatant]/[total absorbance (supernatant + lysate).

### 2.7. Hoechst/PI Staining Assay

Plasma membrane integrity was evaluated using a Hoechst/PI double-staining assay kit (#CA1120, Solarbio) [38]. Cells were seeded in 24-well plates and treated differently. Each well was supplemented with 1 mL staining buffer, 5 µL Hoechst solution, and 5 µL PI solution with a gentle shake. After incubation for 30 min at 4 °C, the plates were rinsed in PBS three times. Representative images were captured using an inverted fluorescence microscope.

### 2.8. Detection of Intracellular ROS Levels

A ROS assay kit (#S0033M, Beyotime) was used to detect intracellular ROS levels. Cells were seeded in 24-well plates and treated differently. Following rinsing in PBS three times, 10 μM DCFH-DA (1 mL/well) was added at 37 °C for 20 min. After another round of rinses, representative images were taken using an inverted fluorescence microscope [40].

### 2.9. Animal Treatment

Twenty male apoE^−/−^ mice (8 weeks old) were bought from Jiangsu Huachuang Sino company (Jiangsu, China). They were kept in an environmentally controlled room (24 ± 2 °C, 60% humidity) and fed an HFD (0.3% cholesterol, 21% fat). Mice were separated into two groups (n = 10 for each group): nicotine group (100 μg/mL of nicotine (#N3876, Sigma-Aldrich) was added to water), and nicotine plus AT-I group (100 μg/mL of nicotine was added to water and 50 mg/kg/d AT-I were administered intragastrically). Sodium carboxymethyl cellulose (0.5%, #9004-32-4, Sigma-Aldrich) was used to dissolve AT-I (#HY-N0201, Medchemexpress). The pentobarbital sodium solution (3%, 30 mg/kg, i.p) was utilized to anesthetize mice. Then, 12 weeks later, mice were euthanized by cervical dislocation. The hearts, aortas, and plasma samples were gathered for further analysis. The Animal Care and Use Committee at Anhui University of Chinese Medicine approved all animal experiments (approval number: AHUCM-mouse-2024223) in line with the NIH Guide for the Care and Use of Laboratory Animals.

### 2.10. Assessment of Atherosclerotic Lesions

Mice were euthanized, and the hearts and aortas were carefully removed. The tissues were thoroughly washed in PBS and soaked in 4% paraformaldehyde overnight. Then, the hearts were cut consecutively using a microtome cryostat (Leica). Serial 6-μm-thick cryosections were obtained (8 sections/mouse). The cross-sectional lesions in the aortic root were stained with Oil Red O (#G1261, Solarbio), HE (#G1120, Solarbio), and Masson (#G1346, Solarbio) to evaluate the lipid accumulation, size of atherosclerotic lesions, collagen contents, and the positively stained regions were outlined using Image Pro Plus software, and the ratio was calculated.

### 2.11. Detection of Serum Biochemical Indices

Blood samples (approximately 1.5 mL per mouse) were collected from the retroorbital plexus under anesthesia and stored in EDTA-coated tubes. Serums (supernatants) were obtained by centrifugation. The levels of aspartate aminotransferase (AST, #C010-2-1), alanine aminotransferase (ALT, #C009-2-1), blood urea nitrogen (BUN, #C013-2-1), serum creatinine (Scr, #C011-2-1, Nanjing Jiancheng Biotech), total cholesterol (TC, #A111-1-1), low-density lipoprotein cholesterol (LDL-C, #A113-1-1), high-density lipoprotein cholesterol (HDL-C, #A112-1-1), and triglycerides (TG, #A110-1-1) were detected per the manufactural instructions (Nanjing Jiancheng Biotech, Jiangsu, China). As mentioned above, IL-1β, IL-6, and TNF-α levels in the serum were detected using ELISA.

### 2.12. Statistical Analyses

All experiments were repeated at least three times, each performed in triplicate. The GraphPad Prism 10.1 software was used to analyze data. The results were presented as mean ± SD. The differences between the two groups were compared using an unpaired two-tailed Student’s t-test. One-way ANOVA followed by Tukey’s multiple comparison test was used to perform multiple comparisons (≥three groups). Statistical significance was taken when *p*  <  0.05.

## 3. Results

### 3.1. Nicotine Promotes NLRP3 Inflammasome Activation and Induces Pyroptosis in THP-1-Derived Macrophages

First, we detected the effects of various doses of nicotine on inflammation and pyroptosis in THP-1-derived macrophages. Based on previous studies [41], cells were treated with nicotine at various concentrations (0 µM, 0.5 µM, and 1 µM) for 24 h. The results showed that nicotine treatment increased the expression of NLRP3, ASC, cleaved caspase-1, and GSDMD-N in a dose-dependent manner (Figure 1B,C). The secretion levels of IL-1β, IL-18, and IL-6, caspase-1 activity, and LDH release were significantly up-regulated (Figure 1D–F). Similarly, the Hoechst/PI staining assay showed a gradual increase in plasma membrane damage in response to nicotine treatment (Figure 1G). These data reveal that nicotine promotes inflammation and pyroptosis in THP-1-derived macrophages.

### 3.2. AT-I Inhibits Nicotine-Induced NLRP3 Inflammasome Activation and Pyroptosis in THP-1-Derived Macrophages

**Figure 2:**
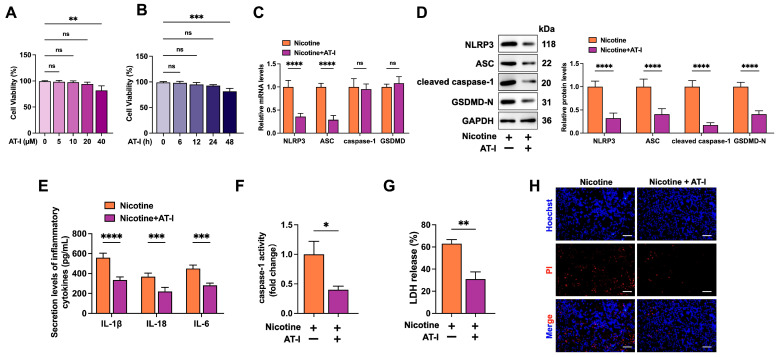
AT-I inhibits nicotine-induced pyroptosis and inflammation in THP-1-derived macrophages. (**A**,**B**) Cells were treated with various doses of AT-I for 24 h or 20 µM AT-I for different durations. Then, cytotoxicity was assessed using the MTT assay. (**C**–**H**) Cells were pretreated with 1 µM nicotine for 24 h, followed by incubation with or without 20 µM AT-I for another 24 h. (**C**) The mRNA levels of pyroptosis-associated factors were determined by RT-qPCR. (**D**) The levels of pyroptosis-associated proteins were detected using Western blot. (**E**) Detection of IL-1β, IL-6, and IL-18 secretion levels by ELISA. (**F**) Detection of caspase-1 activity. (**G**) Detection of LDH release. (**H**) Hoechst/PI staining. Scale bar = 20 μm. * *p* < 0.05, ** *p* < 0.01, *** *p* < 0.001, **** *p* < 0.0001, ns, not significant. *n* = 3.

As shown in Figure 2A,B, macrophages treated with 20 μM AT-I for 24 h did not display toxicity compared to the control group. In addition, compared to the nicotine group, AT-I significantly decreased the levels of pyroptosis-associated factors (Figure 2C,D). Furthermore, AT-I treatment reduced the secretion of pro-inflammatory cytokines (IL-1β, IL-18, and IL-6), decreased the activity of caspase-1, lowered the release of LDH, and improved plasma membrane integrity (Figure 2E–H). These observations demonstrated that AT-I suppresses nicotine-induced pyroptosis and inflammation in THP-1-derived macrophages.

### 3.3. AT-I Inhibits Nicotine-Induced NLRP3 Inflammasome Activation and Pyroptosis in THP-1-Derived Macrophages by Decreasing TXNIP

**Figure 3:**
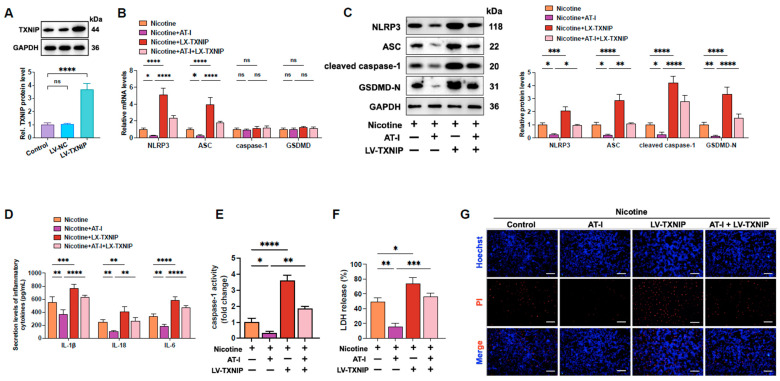
AT-I suppresses nicotine-induced NLRP3 inflammasome activation and pyroptosis of THP-1-derived macrophages by inhibiting TXNIP. (**A**) Cells were transduced with LV-NC or LV-TXNIP for 72 h. Western blot assay was used to detect TXNIP protein levels. (**B**–**G**) Cells were pre-treated with 1 µM nicotine for 24 h. Afterward, cells were transfected with LV-TXNIP and incubated with 20 µM AT-I for 24 h. (**B**) RT-qPCR analysis of the mRNA levels of NLRP3, caspase-1, ASC, and GSDMD. (**C**) Western blot analysis of the protein levels of NLRP3, cleaved caspase-1, ASC, and GSDMD-N. (**D**) Detection of IL-1β, IL-6, and IL-18 secretion levels by ELISA. (**E**) Detection of caspase-1 activity. (**F**) Evaluation of LDH release. (**G**) Hoechst/PI staining. Scale bar = 20 μm. * *p* < 0.05, ** *p* < 0.01, *** *p* < 0.001, **** *p* < 0.0001, ns, not significant. *n* = 3.

TXNIP, a binding partner of thioredoxin (Trx), plays a crucial role in oxidative stress, redox balance, and endoplasmic reticulum stress [42]. TXNIP/NLRP3-signaling-mediated pyroptosis was identified as a critical mechanism in developing CVDs [31,43]. We next investigated whether AT-I inhibited nicotine-induced pyroptosis of THP-1-derived macrophages by blocking TXNIP. Western blot results showed that compared to the control group, transfection with LV-TXNIP significantly increased TXNIP expression by approximately 2.67-fold (Figure 3A). Expectedly, compared to the nicotine plus AT-I group, TXNIP overexpression mainly increased the expression of pyroptosis-associated factors (Figure 3B,C), up-regulated the secretion levels of pro-inflammatory cytokines (Figure 3D), enhanced caspase-1 activity (Figure 3E) and LDH release (Figure 3F), and decreased plasma membrane integrity (Figure 3G). These results demonstrated that AT-I inhibits nicotine-induced pyroptosis by suppressing the TXNIP/NLRP3 pathway in THP-1-derived macrophages.

### 3.4. AT-I Represses Pyroptosis in Nicotine-Treated THP-1-Derived Macrophages by Inhibiting the ROS/TXNIP Pathway

**Figure 4:**
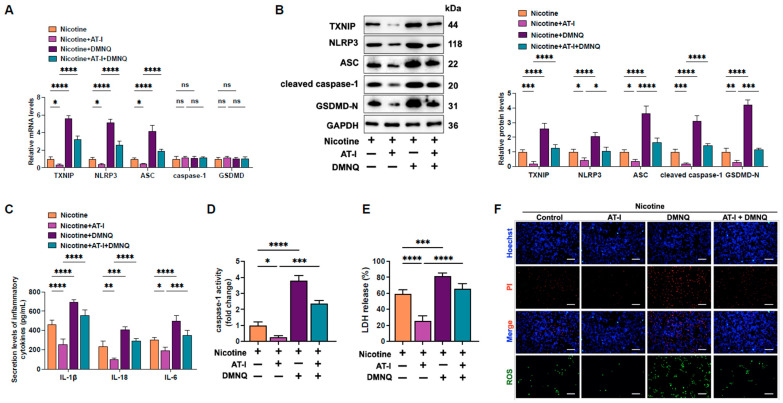
AT-I suppresses nicotine-induced pyroptosis in THP-1-derived macrophages by inhibiting the activation of ROS/TXNIP/NLRP3 signaling. Cells were pre-treated with 1 µM nicotine for 24 h. Then, cells were incubated with 10 µM DMNQ (ROS generator) for 6 h and further incubated with 20 µM AT-I for 24 h. (**A**) RT-qPCR analysis of the mRNA levels of pyroptosis-related factors. (**B**) Western blot analysis of the expression of pyroptosis-related proteins. (**C**) Detection of IL-1β, IL-6, and IL-18 secretion levels by ELISA. (**D**) Detection of caspase-1 activity. (**E**) Evaluation of LDH release. (**F**) Hoechst/PI staining and ROS production. Scale bar = 20 μm. * *p* < 0.05, ** *p* < 0.01, *** *p* < 0.001, **** *p* < 0.0001, ns, not significant. *n* = 3.

Previous research has indicated that ROS are an upstream factor in the TXNIP/NLRP3 signaling pathway-mediated cell pyroptosis [31,44]. We hypothesize that ROS may play a role in the protective effects of AT-I against nicotine-induced pyroptosis in THP-1-derived macrophages. Macrophages were pre-treated with DMNQ (ROS generator) and incubated with AT-I for 24 h. As shown in Figure 4A-F, ROS enhancement reversed the down-regulation of pyroptosis-related factors, the secretion of pro-inflammatory factors, caspase-1 activity, LDH release, and plasma membrane damage induced by AT-I. These results indicated that AT-I alleviates nicotine-induced inflammation and pyroptosis in THP-1-derived macrophages by inhibiting the ROS/TXNIP/NLRP3 pathway.

### 3.5. AT-I Represses Inflammation and Pyroptosis of Nicotine-Treated THP-1-Derived Macrophages Via Inhibition of the TLR4/ROS/TXNIP/NLRP3 Pathway

**Figure 5:**
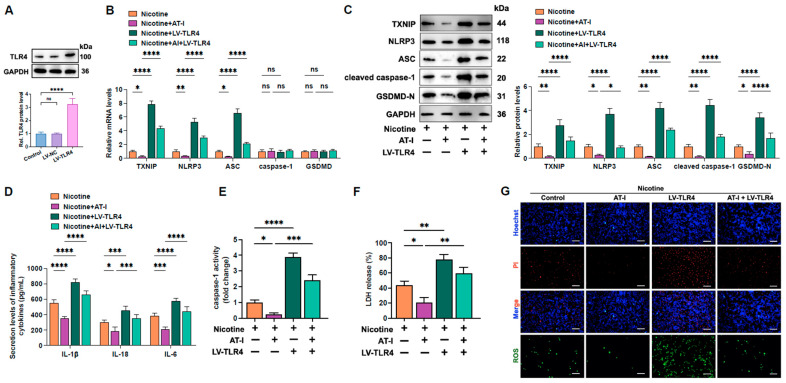
AT-I suppresses the activation of ROS/TXNIP/NLRP3 signaling and pyroptosis in nicotine-treated THP-1-derived macrophages by antagonizing TLR4. (**A**) Cells were transduced with LV-NC or LV-TLR4 for 72 h. Western blot was used to detect TLR4 protein levels. (**B**–**G**) Cells were pre-treated with 1 µM nicotine for 24 h. Afterward, cells were transfected with LV-TLR4 and then incubated with 20 µM AT-I for 24 h. (**B**) RT-qPCR analysis of the mRNA levels of pyroptosis-associated factors. (**C**) Western blot analysis of the levels of pyroptosis-related proteins. (**D**) Detection of IL-1β, IL-6, and IL-18 secretion levels by ELISA. (**E**) Detection of caspase-1 activity. (**F**) Detection of LDH release. (**G**) Hoechst/PI staining and ROS production. Scale bar = 20 μm. * *p* < 0.05, ** *p* < 0.01, *** *p* < 0.001, **** *p* < 0.0001, ns, not significant. *n* = 3.

Previous studies showed that nicotine could exaggerate LPS-induced local airway inflammation by activating TLR4 [45]. AT-I is a novel antagonist of TLR4 [46,47]. We wonder whether AT-I inhibits ROS/TXNIP/NLRP3-mediated pyroptosis and inflammation by suppressing TLR4. Western blot assay showed that compared to the control group, LV-TLR4 treatment increased TLR4 expression by approximately 2.23-fold (Figure 5A). Compared to the nicotine plus AT-I group, TLR4 overexpression robustly increased ROS production and the expression of pyroptosis-related indicators (Figure 5B,C,G). Additionally, the secretion of pro-inflammatory factors, LDH release, caspase-1 activity, and plasma membrane disruption were also amplified (Figure 5D–G), demonstrating that AT-I reduces inflammation and pyroptosis in nicotine-treated THP-1-derived macrophages through inhibition of the TLR4/ROS/TXNIP pathway.

### 3.6. AT-I Mitigates Nicotine-Induced Atherosclerosis in HFD-Fed apoE^−/−^ Mice

**Figure 6:**
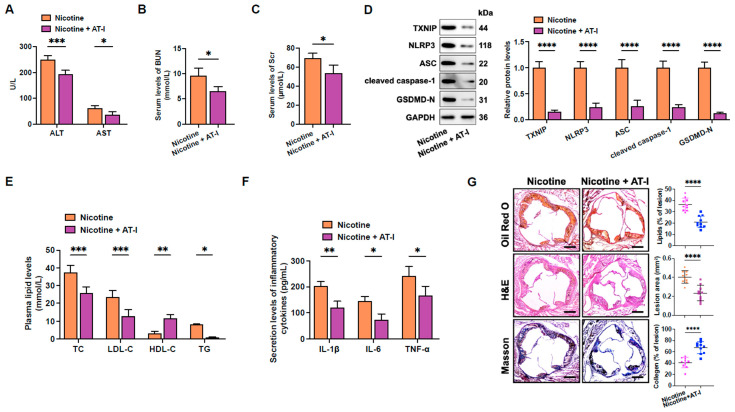
AT-I mitigates the progression of nicotine-induced atherosclerosis in HFD-fed apoE^−/−^ mice. (**A**–**C**) The ALT, AST, BUN, and Scr levels were detected using respective commercial kits; *n* = 3. (**D**) Western blot analyses of pyroptosis-related protein levels in the aorta; *n* = 3. (**E**) Detection of plasma lipid levels; *n* = 5. (**F**) Assessment of pro-inflammatory cytokine levels in the serum; *n* = 5. (**G**) Different staining of the atherosclerotic plaques. Image Pro 7.0 software analyzed the lipid accumulation, lesion area, and collagen content within atherosclerotic plaques. *n* = 10. Scale bar = 100 μm. * *p* < 0.05, ** *p* < 0.01, *** *p* < 0.001, **** *p* < 0.0001, ns, not significant.

Finally, the effects of AT-I on nicotine-induced atherosclerosis were detected. Compared to the nicotine group, AT-I treatment significantly decreased ALT, AST, BUN, and Scr serum levels, suggesting that AT-I can protect against nicotine-induced hepatotoxicity and nephrotoxicity (Figure 6A–C). In addition, AT-I notably decreased the protein levels of pyroptosis-related proteins in the aortas (Figure 6D). The serum levels of TC, LDL-C, TG, IL-6, IL-1β, and TNF-α were down-regulated, while the HDL-C levels were up-regulated in response to AT-I administration (Figure 6E,F). Furthermore, AT-I significantly decreased lipid accumulation in the aortic root. Meanwhile, HE staining indicated a reduction in necrotic core contents. Masson staining showed that AT-I improved plaque stability, as evidenced by increased collagen content (Figure 6G). These findings demonstrate that AT-I inhibits nicotine-induced atherosclerotic plaque development in vivo.

## 4. Discussion

The present study unraveled that AT-I decreases nicotine-induced activation of NLRP3 inflammasome and pyroptosis, reducing macrophages’ pro-inflammatory cytokine secretion. Our data further discovered that AT-I antagonizes TLR4, thereby increasing ROS production. Additionally, we identified that inhibition of the TLR4/ROS pathway served as the upstream signal for suppressing the TXNIP/NLRP3-mediated macrophage pyroptosis. Moreover, it was observed that AT-I improved plasma lipid profile, inhibited inflammatory response, and mitigated the progression of atherosclerotic plaqu+es induced by nicotine in apoE^−/−^ mice.

Accumulating evidence showed that AT-I prevents inflammation and improves lipid metabolism [25,28], whereas its role in atherogenesis has yet to be assessed. Here, we, for the first time, identified that AT-I protects against nicotine-induced atherosclerosis plaque formation, which resulted from the inhibition of macrophage pyroptosis. Several lines of evidence show that nicotine contributes to macrophage pyroptosis through various mechanisms. Hou et al. reported that nicotine could promote pyroptosis of THP-1-derived macrophages via the LINC01272/miR-515/KLF6 axis [48]. Xu et al. found that nicotine treatment increased HDAC6 expression, which activated the NF-κB/NLRP3 pathway, triggering pyroptosis in RAW264.7 macrophages [49]. Cong et al. observed that melatonin alleviated nicotine-induced pyroptosis of THP-1-derived macrophages by regulating the SIRT3/FOXO3α/ROS pathway [41]. In addition, nicotine can promote the pyroptosis of BMDMs by activating the ROS/TXNIP/NLRP3 signaling [29]. Our study discovered that AT-I decreased nicotine-induced ROS production, and pretreatment with ROS agonist or TXNIP overexpression abolished the inhibitory effects of AT-I on NLRP3 inflammasome activation and pyroptosis, demonstrating that AT-I suppresses nicotine-induced macrophage pyroptosis by inhibiting the ROS/TXNIP/NLRP3 pathway. However, whether the LINC01272/miR-515/KLF6 axis, HDAC6/NF-κB/NLRP3 axis, or SIRT3/FOXO3α/ROS pathway is involved in the anti-pyroptotic effects of AT-I warrants further exploration.

AT-I exerts many protective effects by antagonizing TLR4. Du et al. reported that AT-I treatment significantly decreased acetaminophen-induced liver injury and inflammation by inhibiting the TLR4/MAPKs/NF-κB pathways in mice [50]. Long et al. found that AT-I could suppress cell proliferation, migration, and invasion and induce apoptosis of breast cancer cells by inhibiting the TLR4/NF-κB signaling pathway, ameliorating breast cancer tumorigenesis [47]. In addition, AT-I could repress LPS-induced inflammation in RAW264.7 macrophages by inhibiting TLR4 and subsequent activation of ERK1/2, p38, and NF-κB [51]. In our study, we found that TLR4 overexpression contradicted the inhibitory effects of AT-I on ROS/TXNIP/NLRP3 signaling, inflammatory cytokine secretion, and pyroptosis in nicotine-treated THP-1-derived macrophages, indicating that AT-I exerts anti-pyroptotic effects by inhibiting the TLR4/ROS/TXNIP pathway. Further research is needed to determine if MAPK, ERK1/2, p38, and NF-κB are involved in the inhibition of macrophage pyroptosis triggered by AT-I.

Additionally, the ROS/TXNIP/NLRP3 pathway-mediated pyroptosis and inflammation involve several upstream signals, the most significant of which is likely Nrf2. Li et al. found that up-regulation of PPARγ increased Nrf2 expression, which inhibits activation of the ROS/TXNIP/NLRP3 pathway, leading to decreased pyroptosis of HepG2 cells and improved liver dysfunction during sepsis [52]. Zhao et al. unveiled that fructose could decrease miR-200a levels and inhibit Nrf2 expression, which promotes ROS-driven activation of the TXNIP/NLRP3 pathway, resulting in liver inflammation and lipid deposition [53]. Yu et al. observed that Bixin could increase Nrf2 expression, scavenge ROS, and suppress activation of the TXNIP/NLRP3 inflammasome pathway, preventing neuroinflammation and demyelination in mice with experimental autoimmune encephalomyelitis [54]. Importantly, Gao et al. observed that AT-I could inhibit MPTP/MPP^+^-mediated oxidative stress and ROS production in neuroblastoma cells by activating Nrf2, alleviating motor deficits in mice with Parkinson’s disease [55]. It remains to be disclosed, in addition to TLR4, whether AT-I suppresses nicotine-induced ROS/TXNIP/NLRP3 pathway stimulation by activating Nrf2 in THP-1-derived macrophages. Moreover, AT-I has been shown to inhibit macrophage lipid accumulation [25]. Jin et al. reported that incubation of ox-LDL-laden macrophages with caspase-1 inhibitor suppressed pyroptosis and the formation of foam cells [56]. AT-I likely reduces nicotine-induced macrophage lipid accumulation by inhibiting the ROS/TXNIP/NLRP3 pathway-mediated pyroptosis, which requires further investigation for verification.

Additionally, microRNA (miR) plays a crucial role in accelerating the toxicity of cigarette smoking, especially miR-21. Zhu et al. found that nicotine-treated macrophages secreted miR-21-3p-enriched exosomes, which were internalized by vascular smooth muscle cells (VSMCs). Further, miR-21-3p could target phosphatase and tension homologue (PTEN) and promote VSMC proliferation and migration, thereby accelerating the development of atherosclerotic plaques in vivo [57]. miR-21 is aberrantly expressed in many CVDs. In patients with angina or acute myocardial infarction (AMI), plasma miR-21 levels were robustly elevated. In addition, a positive correlation exists between miR-21 and clinically established markers for myocardial necrosis (e.g., cTN and creatine kinase MB) [58]. It is also an early predictor for left ventricular remodeling after myocardial infarction [59]. Importantly, Xue et al. reported that miR-21 could enhance the activation of NF-κB and NLRP3 inflammasome by targeting TNFAIP3, promoting the pyroptosis of BMDMs. In the LPS-treated mice, miR-21 deficiency significantly prevented septic shock and lowered mortality rates [60]. Given the connection among AT-I, nicotine, miR-21, NLRP3, ROS, PTEN, inflammation, and sepsis, we speculate that AT-I may inhibit nicotine-induced septic damage by regulating miR-21, PTEN, and ROS/NLRP3 pathways, which warrants further experiments for verification in the future.

## 5. Conclusions

The present study demonstrates that AT-I protects against nicotine-induced macrophage pyroptosis and atherosclerosis by inhibiting the TLR4/ROS/TXNIP/NLRP3 signaling pathway. AT-I may be an effective herbal medicine for treating atherosclerosis.

## Data Availability

Data are available from the corresponding author upon reasonable request.

## Supplementary Materials

The following supporting information can be downloaded at https://www.mdpi.com/article/10.3390/metabo15050329/s1. Table S1: The sequences of miRNA mimic/inhibitor and PCR primers.

## Abbreviations

AT-I: Atractylenolide I; ASCVD, atherosclerotic cardiovascular disease; IL-1β, interleukin-1β; HFD, high-fat diet; apoE^−/−^, apolipoprotein E deficient; PCD, programmed cell death; GSDM, gasdermin; ROS, reactive oxygen species; HO-1, oxygenase-1; NO, nitric oxide; TNF-α, tumor necrosis factor-alpha; IL-6, interleukin-6; PBMCs, peripheral blood mononuclear cells; BMDMs, bone marrow-derived macrophages; NRF2, NF-E2-related factor 2; LV-NC, lentiviral empty vector; LV-TLR4, lentiviral vector expressing TLR4; MOI, multiplicity of infection; AST, aspartate aminotransferase; ALT, alanine aminotransferase; BUN, blood urea nitrogen; Scr, serum creatinine; TC, total cholesterol; LDL-C, low-density lipoprotein cholesterol; HDL-C, high-density lipoprotein cholesterol; TG, triglyceride; Trx, thioredoxin; miR, microRNA; VSMCs, vascular smooth muscle cells; PTEN, phosphatase and tension homologue; AMI, acute myocardial infarction.

## References

[1] Rosenblit P.D. (2019). Extreme Atherosclerotic Cardiovascular Disease (ASCVD) Risk Recognition. Curr. Diab Rep..

[2] Adkar S.S., Leeper N.J. (2024). Efferocytosis in atherosclerosis. Nat. Rev. Cardiol..

[3] Svensson E.C., Madar A., Campbell C.D., He Y., Sultan M., Healey M.L., Xu H., D’Aco K., Fernandez A., Wache-Mainier C. (2022). TET2-Driven Clonal Hematopoiesis and Response to Canakinumab: An Exploratory Analysis of the CANTOS Randomized Clinical Trial. JAMA Cardiol..

[4] Paramel Varghese G., Folkersen L., Strawbridge R.J., Halvorsen B., Yndestad A., Ranheim T., Krohg-Sørensen K., Skjelland M., Espevik T., Aukrust P. (2016). NLRP3 Inflammasome Expression and Activation in Human Atherosclerosis. J. Am. Heart Assoc..

[5] Wang M., Yu F., Zhang Y., Li P. (2023). Programmed cell death in tumor immunity: Mechanistic insights and clinical implications. Front. Immunol..

[6] Lyu T., Yin Q. (2023). Research Progress on Pyroptosis in Hematological Malignancies. Curr. Treat. Options Oncol..

[7] Duewell P., Kono H., Rayner K.J., Sirois C.M., Vladimer G., Bauernfeind F.G., Abela G.S., Franchi L., Nuñez G., Schnurr M. (2010). NLRP3 inflammasomes are required for atherogenesis and activated by cholesterol crystals. Nature.

[8] Fan X., Han J., Zhong L., Zheng W., Shao R., Zhang Y., Shi S., Lin S., Huang Z., Huang W. (2024). Macrophage-Derived GSDMD Plays an Essential Role in Atherosclerosis an d Cross Talk Between Macrophages via the Mitochondria-STING-IRF3/NF-κB Axis. Arterioscler. Thromb. Vasc. Biol..

[9] Weng X., Luo X., Dai X., Lv Y., Zhang S., Bai X., Bao X., Wang Y., Zhao C., Zeng M. (2023). Apigenin inhibits macrophage pyroptosis through regulation of oxidativ e stress and the NF-κB pathway and ameliorates atherosclerosis. Phytother. Res..

[10] Shao X., Zeng W., Wang Q., Liu S., Guo Q., Luo D., Luo Q., Wang D., Wang L., Zhang Y. (2024). Fufang Zhenzhu Tiaozhi (FTZ) suppression of macrophage pyroptosis: Key to stabilizing rupture-prone plaques. J. Ethnopharmacol..

[11] Pan X., Xu H., Ding Z., Luo S., Li Z., Wan R., Jiang J., Chen X., Liu S., Chen Z. (2024). Guizhitongluo Tablet inhibits atherosclerosis and foam cell formation through regulating Piezo1/NLRP3 mediated macrophage pyroptosis. Phytomedicine.

[12] World Health Organization (2017). WHO Report on the Global Tobacco Epidemic 2017: Monitoring Tobacco Use And prevention Policies.

[13] World Health Organization (2011). WHO Report on the Global Tobacco Epidemic, 2011: Warning About the Dangers of Tobacco.

[14] Fu X., Zong T., Yang P., Li L., Wang S., Wang Z., Li M., Li X., Zou Y., Zhang Y. (2021). Nicotine: Regulatory roles and mechanisms in atherosclerosis progressi on. Food Chem. Toxicol..

[15] Deng M., Chen H., Long J., Song J., Xie L., Li X. (2021). Atractylenolides (I, II, and III): A review of their pharmacology and pharmacokinetics. Arch. Pharm. Res..

[16] Zhu B., Zhang Q.L., Hua J.W., Cheng W.L., Qin L.P. (2018). The traditional uses, phytochemistry, and pharmacology of Atractylodes macrocephala Koidz.: A review. J. Ethnopharmacol..

[17] Wang C.C., Lin S.Y., Cheng H.C., Hou W.C. (2006). Pro-oxidant and cytotoxic activities of atractylenolide I in human promyeloleukemic HL-60 cells. Food Chem. Toxicol..

[18] Fu X.Q., Chou J.Y., Li T., Zhu P.L., Li J.K., Yin C.L., Su T., Guo H., Lee K.W., Hossen M.J. (2018). The JAK2/STAT3 pathway is involved in the anti-melanoma effects of atractylenolide I. Exp. Dermatol..

[19] Li Y., Wang Y., Liu Z., Guo X., Miao Z., Ma S. (2020). Atractylenolide I Induces Apoptosis and Suppresses Glycolysis by Blocking the JAK2/STAT3 Signaling Pathway in Colorectal Cancer Cells. Front. Pharmacol..

[20] Yu R., Yu B.-x., Chen J.-f., Lv X.-y., Yan Z.-j., Cheng Y., Ma Q. (2016). Anti-tumor effects of Atractylenolide I on bladder cancer cells. J. Exp. Clin. Cancer Res..

[21] Sun C., Zhang X., Yu F., Liu C., Hu F., Liu L., Chen J., Wang J. (2021). Atractylenolide I alleviates ischemia/reperfusion injury by preserving mitochondrial function and inhibiting caspase-3 activity. J. Int. Med. Res..

[22] Chen S., Liu X., Zhou X., Lin W., Liu M., Ma H., Zhong K., Ma Q., Qin C. (2025). Atractylenolide-I prevents abdominal aortic aneurysm formation through inhibiting inflammation. Front. Immunol..

[23] Zhou Z.Y., Ma J., Zhao W.R., Shi W.T., Zhang J., Hu Y.Y., Yue M.Y., Zhou W.L., Yan H., Tang J.Y. (2024). Qiangxinyin formula protects against isoproterenol-induced cardiac hypertrophy. Phytomedicine.

[24] Zhang J.-l., Huang W.-m., Zeng Q.-y. (2015). Atractylenolide I protects mice from lipopolysaccharide-induced acute lung injury. Eur. J. Pharmacol..

[25] Li W., Zhi W., Liu F., He Z., Wang X., Niu X. (2017). Atractylenolide I restores HO-1 expression and inhibits Ox-LDL-induced VSMCs proliferation, migration and inflammatory responses in vitro. Exp. Cell Res..

[26] Wang C., Duan H., He L. (2009). Inhibitory effect of atractylenolide I on angiogenesis in chronic infl ammation in vivo and in vitro. Eur. J. Pharmacol..

[27] Wang D., Lin Z., Zhou Y., Su M., Zhang H., Yu L., Li M. (2024). Atractylenolide I ameliorates sepsis-induced cardiomyocyte injury by i nhibiting macrophage polarization through the modulation of the PARP1/ NLRP3 signaling pathway. Tissue Cell.

[28] Li C.-Q., He L.-C., Jin J.-Q. (2007). Atractylenolide I and atractylenolide III inhibit Lipopolysaccharide-i nduced TNF-alpha and NO production in macrophages. Phytother. Res..

[29] Mao C., Li D., Zhou E., Zhang J., Wang C., Xue C. (2021). Nicotine exacerbates atherosclerosis through a macrophage-mediated end othelial injury pathway. Aging.

[30] Cui Y., Chen X.-B., Liu Y., Wang Q., Tang J., Chen M.-J. (2024). Piperlongumine inhibits esophageal squamous cell carcinoma in vitro and in vivo by triggering NRF2/ROS/TXNIP/NLRP3-dependent pyroptosis. Chem. Biol. Interact..

[31] Wang Y., Yuan H., Shen D., Liu S., Kong W., Zheng K., Yang J., Ge L. (2024). Artemisinin attenuated ischemic stroke induced pyroptosis by inhibiting ROS/TXNIP/NLRP3/Caspase-1 signaling pathway. Biomed. Pharmacother..

[32] Wang Y., Wang J., Chen Y., Li X., Jiang Z. (2025). Decursinol angelate relieves inflammatory bowel disease by inhibiting the ROS/TXNIP/NLRP3 pathway and pyroptosis. Front. Pharmacol..

[33] Liang J., Lin X., Jiang C., Liu Y., Hao Z., Qiu M., Liu X., Chen D., Teng X., Tang Y. (2024). Molecular mechanism of apoptosis induced by 4-tBP in common carp (*Cyprinus carpio* L.) head kidneys was explored from various angles: Hippo pathway, miR-203a, oxidative stress, ER stress, and mitochondrial pathway. Aquaculture.

[34] Wang S., Li X., Zhang M., Qian Y., Li E., Teng X., Li M. (2024). miR-199-5p mediates the regulation of autophagy by targeting mTOR signaling and involvement in ammonia detoxification under ammonia stress in yellow catfish (*Pelteobagrus fulvidraco*). Aquaculture.

[35] Che X., Shang X., WeiXu, Xing M., Wei H., Li W., Li Z., Teng X., Geng L. (2025). Selenium-enriched Lactiplantibacillus plantarum alleviates alkalinity stress-induced selective hepatic insulin resistance in common carp. Int. J. Biol. Macromol..

[36] Hao Z., Qiu M., Liu Y., Liu Y., Chang M., Liu X., Wang Y., Sun W., Teng X., Tang Y. (2025). Co-exposure to ammonia and lipopolysaccharide-induced impaired energy metabolism via the miR-1599/HK2 axis and triggered autophagy, ER stress, and apoptosis in chicken cardiomyocytes. Poult. Sci..

[37] Zhou Q., Hao Z., Qiu M., Liu Y., Chang M., Liu X., Wang Y., Tang Y., Sun W., Teng X. (2025). Amino acid metabolism disorder and oxidative stress took part in EGCG alleviating Mn-caused ferroptosis via miR-9-5p/got1 axis. J. Hazard. Mater..

[38] Liang Y., Xu X.D., Xu X., Cai Y.B., Zhu Z.X., Zhu L., Ren K. (2023). Linc00657 promoted pyroptosis in THP-1-derived macrophages and exacerbated atherosclerosis via the miR-106b-5p/TXNIP/NLRP3 axis. Int. J. Biol. Macromol..

[39] Xie X., Zhao Y., Ma C.Y., Xu X.M., Zhang Y.Q., Wang C.G., Jin J., Shen X., Gao J.L., Li N. (2015). Dimethyl fumarate induces necroptosis in colon cancer cells through GSH depletion/ROS increase/MAPKs activation pathway. Br. J. Pharmacol..

[40] Qiu M., Hao Z., Liu Y., Liu Y., Chang M., Lin X., Liu X., Dong N., Sun W., Teng X. (2025). ROS acted as an initial role in selenium nanoparticles alleviating insecticide chlorpyrifos-induced oxidative stress, pyroptosis, and intestinal barrier dysfunction in porcine intestinal epithelial cells. Pestic. Biochem. Physiol..

[41] Cong L., Liu X., Bai Y., Qin Q., Zhao L., Shi Y., Bai Y., Guo Z. (2023). Melatonin alleviates pyroptosis by regulating the SIRT3/FOXO3α/ROS axis and interacting with apoptosis in Atherosclerosis progression. Biol. Res..

[42] Choi E.-H., Park S.-J. (2023). TXNIP: A key protein in the cellular stress response pathway and a potential therapeutic target. Exp. Mol. Med..

[43] Chen M., Wang R., Liao L., Li Y., Sun X., Wu H., Lan Q., Deng Z., Liu P., Xu T. (2024). DanShen Decoction targets miR-93-5p to provide protection against MI/RI by regulating the TXNIP/NLRP3/Caspase-1 signaling pathway. Phytomedicine.

[44] Gu C., Draga D., Zhou C., Su T., Zou C., Gu Q., Lahm T., Zheng Z., Qiu Q. (2019). miR-590-3p Inhibits Pyroptosis in Diabetic Retinopathy by Targeting NLRP1 and Inactivating the NOX4 Signaling Pathway. Investig. Ophthalmol. Vis. Sci..

[45] Xu Y., Zhang Y., Cardell L.O. (2014). Nicotine exaggerates LPS-induced airway hyperreactivity via JNK-mediated up-regulation of Toll-like receptor 4. Am. J. Respir. Cell Mol. Biol..

[46] Liu H., Zhang G., Huang J., Ma S., Mi K., Cheng J., Zhu Y., Zha X., Huang W. (2016). Atractylenolide I modulates ovarian cancer cell-mediated immunosuppression by blocking MD-2/TLR4 complex-mediated MyD88/NF-κB signaling in vitro. J. Transl. Med..

[47] Long F., Lin H., Zhang X., Zhang J., Xiao H., Wang T. (2020). Atractylenolide-I Suppresses Tumorigenesis of Breast Cancer by Inhibiting Toll-Like Receptor 4-Mediated Nuclear Factor-κB Signaling Pathway. Front. Pharmacol..

[48] Hou L., He Q., Wang Y., Feng X., Mi Y., Li S., Deng J.F., Zhao G. (2023). Nicotine induces macrophage pyroptosis via LINC01272/miR-515/KLF6 axis. Ecotoxicol. Environ. Saf..

[49] Xu S., Chen H., Ni H., Dai Q. (2021). Targeting HDAC6 attenuates nicotine-induced macrophage pyroptosis via NF-κB/NLRP3 pathway. Atherosclerosis.

[50] Du Z., Ma Z., Lai S., Ding Q., Hu Z., Yang W., Qian Q., Zhu L., Dou X., Li S. (2022). Atractylenolide I Ameliorates Acetaminophen-Induced Acute Liver Injury via the TLR4/MAPKs/NF-κB Signaling Pathways. Front. Pharmacol..

[51] Ji G., Chen R., Zheng J. (2014). Atractylenolide I inhibits lipopolysaccharide-induced inflammatory responses via mitogen-activated protein kinase pathways in RAW264.7 cells. Immunopharmacol. Immunotoxicol..

[52] Li Z., Liu T., Feng Y., Tong Y., Jia Y., Wang C., Cui R., Qu K., Liu C., Zhang J. (2022). PPARγ Alleviates Sepsis-Induced Liver Injury by Inhibiting Hepatocyte Pyroptosis via Inhibition of the ROS/TXNIP/NLRP3 Signaling Pathway. Oxid. Med. Cell Longev..

[53] Zhao X.J., Yu H.W., Yang Y.Z., Wu W.Y., Chen T.Y., Jia K.K., Kang L.L., Jiao R.Q., Kong L.D. (2018). Polydatin prevents fructose-induced liver inflammation and lipid deposition through increasing miR-200a to regulate Keap1/Nrf2 pathway. Redox Biol..

[54] Yu Y., Wu D.M., Li J., Deng S.H., Liu T., Zhang T., He M., Zhao Y.Y., Xu Y. (2020). Bixin Attenuates Experimental Autoimmune Encephalomyelitis by Suppressing TXNIP/NLRP3 Inflammasome Activity and Activating NRF2 Signaling. Front. Immunol..

[55] Gao Y., Li S., Zhang S., Zhang Y., Zhang J., Zhao Y., Chang C., Gao X., Chen L., Yang G. (2024). Atractylenolide-I Attenuates MPTP/MPP(+)-Mediated Oxidative Stress in Parkinson’s Disease Through SIRT1/PGC-1α/Nrf2 Axis. Neurochem. Res..

[56] Jin Y., Liu Y., Xu L., Xu J., Xiong Y., Peng Y., Ding K., Zheng S., Yang N., Zhang Z. (2022). Novel role for caspase 1 inhibitor VX765 in suppressing NLRP3 inflammasome assembly and atherosclerosis via promoting mitophagy and efferocytosis. Cell Death Dis..

[57] Zhu J., Liu B., Wang Z., Wang D., Ni H., Zhang L., Wang Y. (2019). Exosomes from nicotine-stimulated macrophages accelerate atherosclerosis through miR-21-3p/PTEN-mediated VSMC migration and proliferation. Theranostics.

[58] Kabłak-Ziembicka A., Badacz R., Przewłocki T. (2022). Clinical Application of Serum microRNAs in Atherosclerotic Coronary Artery Disease. J. Clin. Med..

[59] Liu X., Dong Y., Chen S., Zhang G., Zhang M., Gong Y., Li X. (2015). Circulating MicroRNA-146a and MicroRNA-21 Predict Left Ventricular Remodeling after ST-Elevation Myocardial Infarction. Cardiology.

[60] Xue Z., Xi Q., Liu H., Guo X., Zhang J., Zhang Z., Li Y., Yang G., Zhou D., Yang H. (2019). miR-21 promotes NLRP3 inflammasome activation to mediate pyroptosis and endotoxic shock. Cell Death Dis..

