# Supplementary material for: Atractylenolide I Inhibits Nicotine-Induced Macrophage Pyroptosis and Alleviates Atherogenesis by Suppressing the TLR4/ROS/TXNIP/NLRP3 Pathway
Source: Metabolites. 2025 May 15;15(5):329. doi: 10.3390/metabo15050329 (PMC12114077; doi:10.3390/metabo15050329)
Supplement: Supplementary file 1 [file metabolites-15-00329-s001.zip › metabolites-3584345-supplementary.pdf]

Supplementary Table S1. The sequences of miRNA mimic/inhibitor and PCR primers.

TLR4 (Human)	Forward, 5'-CCCTGAGGCATTTAGGCAGCTA-3';<br>Reverse, 5'-AGGTAGAGAGGTGGCTTAGGCT-3'
TXNIP (Human)	Forward, 5'-CAGCAGTGCAAACAGACTTCGG-3';<br>Reverse, 5'-CTGAGGAAGCTCAAAGCCGAAC-3'
NLRP3 (Human)	Forward, 5'-GGACTGAAGCACCTGTTGTGCA-3';<br>Reverse, 5'-TCCTGAGTCTCCCAAGGCATTC-3'
Caspase-1 (Human)	Forward, 5'-GCTGAGGTTGACATCACAGGCA-3';<br>Reverse, 5'-TGCTGTCAGAGGTCTTGTGCTC-3'
ASC (Human)	Forward, 5'-AGCTCACCGCTAACGTGCTGC-3';<br>Reverse, 5'-GCTTGGCTGCCGACTGAGGAG-3'
GSDMD (Human)	Forward, 5'-ATGAGGTGCCTCCACAACTTCC-3';<br>Reverse, 5'-CCAGTTCCTTGGAGATGGTCTC-3'
GAPDH (Human)	Forward, 5'-GTCTCCTCTGACTTCAACAGCG-3';<br>Reverse, 5'-ACCACCCTGTTGCTGTAGCCAA-3'
